# Codon usage variability determines the correlation between proteome and transcriptome fold changes

**DOI:** 10.1186/1752-0509-5-33

**Published:** 2011-02-25

**Authors:** Roberto Olivares-Hernández, Sergio Bordel, Jens Nielsen

**Affiliations:** 1 Systems Biology, Department of Chemical and Biological Engineering, Chalmers University of Technology, Kemivägen 10, Gothenburg, SE-41296, Sweden

## Abstract

**Background:**

The availability of high throughput experimental methods has made possible to observe the relationships between proteome and transcirptome. The protein abundances show a positive but weak correlation with the concentrations of their cognate mRNAs. This weak correlation implies that there are other crucial effects involved in the regulation of protein translation, different from the sole availability of mRNA. It is well known that ribosome and tRNA concentrations are sources of variation in protein levels. Thus, by using integrated analysis of omics data, genomic information, transcriptome and proteome, we aim to unravel important variables affecting translation.

**Results:**

We identified how much of the variability in the correlation between protein and mRNA concentrations can be attributed to the gene codon frequencies. We propose the hypothesis that the influence of codon frequency is due to the competition of cognate and near-cognate tRNA binding; which in turn is a function of the tRNA concentrations. Transcriptome and proteome data were combined in two analytical steps; first, we used Self-Organizing Maps (SOM) to identify similarities among genes, based on their codon frequencies, grouping them into different clusters; and second, we calculated the variance in the protein mRNA correlation in the sampled genes from each cluster. This procedure is justified within a mathematical framework.

**Conclusions:**

With the proposed method we observed that in all the six studied cases most of the variability in the relation protein-transcript could be explained by the variation in codon composition.

## Background

The integration of large scale transcriptome and proteome data along with genome-wide sequence information can give insights into the molecular mechanisms that control cellular functions. Moreover, formulation of mathematical models, either mechanistic or statistic, to express such molecular mechanisms remains a challenging task to understand system properties [1]. The correlation between mRNA transcripts and their corresponding cognate proteins has been found to be positive, but it is not sufficiently good to predict protein levels based on their cognate transcript [2,3]. If all the mRNAs were translated at a constant rate the correlation between mRNA and protein concentration would be high. The observed lack of correlation is therefore due to the particularities of the translation mechanism. For instance, in yeast 73% of the variance in protein abundance is explained by the translation mechanism and only 27% due to the variations of the mRNA concentration [4,5]. To explain the differences in the responses between protein and transcript levels recent studies attempted to include information of the translation mechanism by using mechanistic modeling [6] or by using DNA sequence variables and statistic modeling [7]. Several publications have focused on the kinetics of translation; consisting of initiation, elongation and termination phases. For instance, using a gene-sequence-specific mechanistic model, Mehra and Hatzimanikatis [8] studied the rates of initiation, elongation and termination and found that the different response to mRNA levels is mainly dependent on the initiation step. Following these results, Zouridis and Hatzimanikatis [9] suggested that maximization of translation rate can be achieved by an interplay between ribosomal occupancy and ribosome distribution along the translated mRNA fragment. Subsequently, in a following study by the same authors [10], it was found that not only initiation is a controlling step, but also the elongation phase, which is function of the of tRNA concentration. The mentioned authors reformulated their mathematical model to include the competition between the different aminoacyl-tRNA's.

Codon usage has been shown to be correlated with the abundance of transcripts and proteins [11]. Sharp and Li [12] observed that the variability in mRNA levels of different genes is related to their codon usage and the genome-wide codon usage is related to the number of copies of tRNA genes [13]. Recent studies in E. coli have demonstrated experimentally that perturbation in the codon usage of a set of 40 proteins affected both the translation of the proteins and the tRNA levels in the cell [14].

Based on the analysis of published experimental proteome and transcriptome data for the yeast *Saccharomyces cerevisiae *(Additional file 1) we tried to evaluate how much the variance in the protein-mRNA correlation is affected by differences in codon usage; which has been demonstrated to be a relevant factor that affects the translation efficiency, either, by increasing the proofreading efficiency of the codon or modifying the folding energy of the mRNA [15,16]. The protein datasets used in this analysis are the result of experimental setups to quantify the peptides associated to each protein, therefore these techniques account for the amount of translated protein and, as it was suggested by Greenbaum et al [17], the protein level can be defined as the "translatome".

## Methods

### Molecular mechanisms of translation

**Figure 1:**
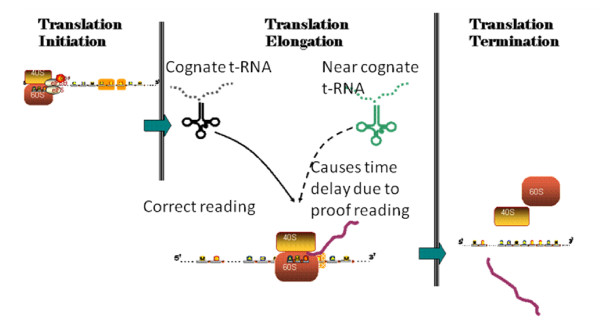
**Translation of mRNA into proteins consists in three steps, initiation, elongation and termination**. The elongation process consists in the attachment of the cognate tRNA in the right sequence position. Due to Wobble interactions near cognates compete for the position in the ribosome site A causing a delay in elongation time.

Translation in yeast starts by the formation of the PIC (pre-initiation complex) which is formed in three steps: first, binding of the specific initiation Met-tRNA to the small ribosomal subunit; second, the resulting complex binds to the mRNA molecules localizing the start codon; and third, the attachment of large ribosomal subunit to generate the polysome structure. All these events are assisted by cis-acting proteins called translation factors. For the elongation process the polysome structure generates three binding sites (E,P,A). In each step an AA-tRNA has to reach the position of site A to place the correct amino acid in the peptide sequence [18,19]. Nevertheless, the existing wobble interactions generate a competition between the cognate and near cognates of charged tRNA (AA-tRNA). Thus, the elongation rate is the result of the time needed to transport the cognate AA-tRNA molecule to the site A in the ribosome [20]. As this is not an efficiently selective step, near cognates can interact in place causing delay due to proof reading and rejection (Figure 1).

### Mathematical framework

**Figure 2:**
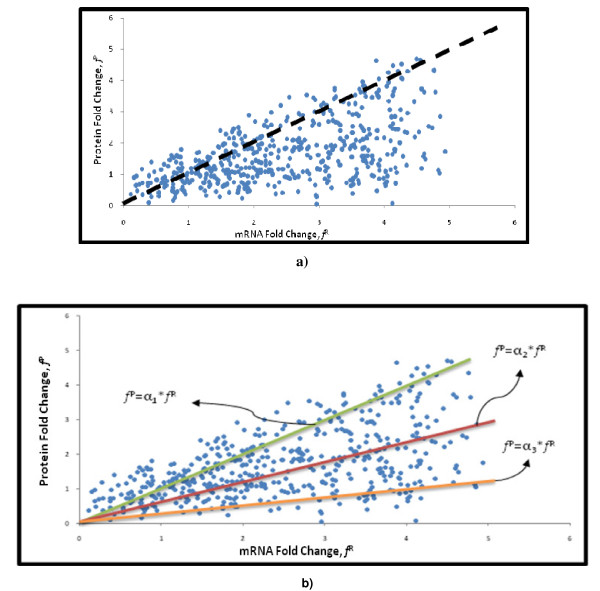
**Transcriptome and proteome correlations**. a) the plot presents transcriptome and proteome experimental data where it is observed that there is a substantial deviation from the correlation one-to-one represented by the dashed line; b) the relationship between proteome and transcriptome is a function of the amplification factor α which accounts for different parameters such, tRNA availability, ribosome density, protein and transcript degradation rates, among others.

Conceptually there is a remarkable difference between correlating abundance expressed in molecules per cell units compared to fold change in abundance. For our analysis we have collected six datasets where fold changes were studied. For instance, in Figure 2a), the plot contains the values of protein and mRNA fold changes for different genes. If the protein concentration were proportional to mRNA concentration, the fold changes (*f*_*j*_) between conditions should be equal:

(1) $$f_{j}^{P}=f_{j}^{R}$$

for *j *= 1...number of genes in the dataset. The superscript P and R correspond to Protein and mRNA quantities, respectively. If such relation were true, the experimental values should fall along the dashed line which is the one-to-one relationship, Figure 2a). If the proportionality constant between mRNA and protein concentrations changed between conditions, the expected graph would be a straight line with slope different from one. However what we found experimentally is a set of scattered points. This means that the proportionality constant not only changes between conditions but also does it differently for each protein.

(2) $$f_{j}^{P}=\alpha_{j}f_{j}^{R}$$

where the constant α can take different positive values; plot b) in Figure 2. This constant can be seen as an amplification factor that implicitly contains the variation from different sources such as: posttranscriptional events, modification in the translation rates and protein half-lives.

The differential equation governing the concentration of a particular protein is the following one [21-23]:

(3) $$\frac{d{\left[P\right]}_{j}}{dt}=k_{s,j}{\left[mRNA\right]}_{j}-k_{d,j}{\left[P\right]}_{j}-\mu {\left[P\right]}_{j}$$

Where [P] is the concentration of each protein, [mRNA] is the concentration of mRNA, ***k***_***s,j ***_and ***k***_***d***__j _are the protein synthesis and degradation rate constants; the dilution term is equal to the growth rate ***μ***. In our approach we write the constant *k*_*s,j *_as the ratio of two characteristic parameters, the number of ribosomes united to each mRNA molecule ***ρ***_***Rj ***_and the elongation time of the protein *t*_*j*_. Note that this substitution is absolutely rigorous. The number of proteins synthesized per unit of time is equal to the number of ribosomes synthesizing the corresponding protein divided by the time that each ribosome takes to synthesize a protein.

(4) $$\frac{d{\left[P\right]}_{j}}{dt}=\frac{\rho_{Rj}}{t_{j}}{\left[mRNA\right]}_{j}-k_{d,j}{\left[P\right]}_{j}-\mu {\left[P\right]}_{j}$$

The two negative terms in the equation correspond to the degradation rate and dilution of proteins as a result of the cellular growth. On the other hand, the elongation time depends on the gene codon composition in the following way

(5) $$t_{j}=\sum_{i}S_{ij}\tau_{i}$$

Where ***S***_***ij ***_is the number of codons *i *in the gene *j *and ***τ***_***i ***_is the average time that will take to add the corresponding amino acid to the nascent peptide. This average time is specific for each codon and it depends on the concentration of the corresponding tRNA. The lower is the concentration of a particular tRNA, the longer the time that it takes to add it. The specific time also increases with the number of wrong proof readings that the ribosome performs before adding the right tRNA [20,24].

Assuming steady state for each protein and supposing that only the elongation time changes between proteins and all the other parameters can change in between conditions but not between proteins, we obtained the following relation between mRNA and protein fold changes.

(7) $$f_{j}^{P}=CT_{j}f_{j}^{R}$$

Where the non-dimensional groups are,

(8) $$C=\frac{\frac{\rho_{R}^{2}}{\rho_{R}^{1}}}{\frac{k_{d}^{2}+\mu^{2}}{k_{d}^{1}+\mu^{1}}};\;T_{j}=\frac{t_{j}^{1}}{t_{j}^{2}}=\frac{\sum_{i}S_{ij}\tau_{i}^{1}}{\sum_{i}S_{ij}\tau_{i}^{2}};\;f_{j}^{P}=\frac{{\left[P\right]}_{j}^{2}}{{\left[P\right]}_{j}^{1}};\;f_{j}^{R}=\frac{{\left[mRNA\right]}_{j}^{2}}{{\left[mRNA\right]}_{j}^{1}}$$

The factor T_j _depends on the protein composition and the tRNA concentrations in each of the two compared conditions, while the factor C groups all the effects that have been considered to vary only between conditions and do not depend on the protein. If this hypothesis were true, the genes with similar codon frequencies would show a similar behavior in their relation between protein and mRNA fold changes.

### Clustering

In this paper we want to evaluate the effects of the codon frequency on protein translation. Proteins with similar codon contents (S_ij_) will have similar values for the coefficient T_j_, if our hypothesis is correct, in a cluster of proteins with similar T_j _the variability of the ratio f_j_^P^/f_j_^R ^will be smaller than in the full proteome. We clustered genes using information about the codon composition which was extracted from the genome sequence downloaded from SGD (http://www.yeastgenome.org/). The codon usage has already been shown to be one of the sequence features most highly associated with protein expression [14,25]. The data were normalized using the total codon content of each gene (Σ_i_S_ij_).

**Figure 3:**
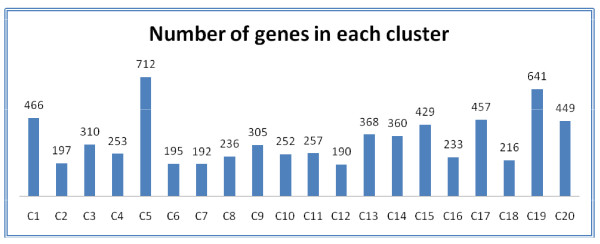
**Using the genome amino acid sequence content from yeast and applying SOM analysis, the result shows 20 different clusters with different numbers of ORFs**.

To cluster the proteins according to the codon usage data we used an unsupervised clustering method analysis, SOM, which is a clustering method based on neural networks, and it helps to visualize datasets by mapping a high dimensional data space into a two dimensional space [26]. SOM analysis provides a robust clustering method for outliers or data dispersion [27,28]. There is no theoretical background that dictates the number of map units (neurons) to build the grid; therefore we selected 20 units as it gave the best distribution of genes across the clusters (see Figure 3).

### GO enrichment analysis

To elucidate if the genes in each cluster shown functional enrichment we performed a Gene Ontology (GO) enrichment analysis. We performed hypergeometric tests using GO functional annotation from SGD to identify which GO biological process terms are enriched in each category. GO enrichment analysis was performed using BINGO tool [29]; a Cytoscape plug in. To identify which GO terms where significant we used a p-value less that 0.01 as a cutoff.

### Analysis of variance

For each of the clusters obtained from the SOM analysis we calculated the ratio between the fold changes in transcriptome and proteome obtaining the value of α and applied the log2 transformation. Logarithmic transformation of data is commonly used as this transformation tends to provide values that are approximately normally distributed and for which ANOVA tests are appropriate [30]. Box plots and histograms showing the distribution of the data are in Additional File 2.

This was done for each protein within each cluster. The subsequent statistical tests will be performed on the following random variable:

(9) $$x_{j}=log_{2}\frac{f_{j}^{P}}{f_{j}^{R}}$$

ANOVA is a hypothesis test method suitable to compare the means across different groups; clusters in our case. Nevertheless, in this study we focus on quantifying the variance inside the clusters compared with the variance in the complete dataset. In this manner, the results will shed light on the amount of variance in expression levels due effects of the codon frequency and the associated tRNA competition in each of the different clusters. To calculate how much of the total variance for the whole data set was observed between clusters and within clusters the following mathematical formalism is needed. The total sum of squares is the sum of the squares within each cluster plus the sum of squares between the clusters.

(10) $$SS_{Total}=SS_{between}+SS_{within}$$

Where:

(11) $$SS_{within}=\sum_{c}{\left(\sum_{j}x_{jc}-{\bar{x}}_{c}\right)}^{2}$$

and

(12) $$SS_{between}=\sum_{c}n_{c}{\left({\bar{x}}_{c}-\bar{x}\right)}^{2}$$

The index *j *identifies each protein inside a given cluster and the index *c *identifies each cluster. The number of proteins in cluster *c *is noted as *n*_*c*_. The main question we are trying to answer is how much of the experimental variation in the fold changes can be explained by the variation in codon frequencies. The rest of the variation will be the result of changes in parameters such as degradation rate or number of ribosomes per mRNA molecule that we have grouped in the factor ***C ***in Eq.7.

### Experimental data

We used six experimental datasets on transcriptome and proteome sampling of the yeast *S. cerevisiae*. All datasets were collected from the literature and each of them involves a different kind of cellular perturbation. To identify each of the datasets we used an ID which is composed using the last name of the first author: i.e, Griffin [31], Ideker [32], and Washburn [33]. For the dataset of Usaite [34,35] the ID is further specifying the type of deletion performed; e.g.Usaite.snf1 is the ID for deletion of the *SNF1 *gene in their study. The details for each dataset are presented in Additional File 1 (supplementary table S1). These data consist of fold change values, differently from other studies that have used abundance (molecules/cell) [36] to study the correlation between protein and mRNA and the co-variables that affect such correlation [15,37]. In a similar approach, Nie et al 2006 [38,39] used fold change ratios to demonstrate the correlation between mRNA and protein expression.

## Results and Discussion

Correlation between proteome and transcriptome abundance in yeast has been widely studied and it has been observed to be weakly positive [2,3]. Fold changes have shown weak positive correlations as well [31]. In this analysis we used experimental transcriptome and proteome data from yeast (See table in Additional File 1 for more details) to investigate how much of the variance in the relationship between these two quantities is explained by the variance in codon usage [14,15,25,40,41]. More details of the experimental techniques of the datasets shown in Additional File 1 (supplementary table S2) can be seen elsewhere [31-35]. It has been demonstrated by Najafabadi et al. [14] that the codon usage content provides direct information about the translation elongation rate based on the demand of tRNA, which affects the fold change of the protein levels. Nevertheless, there are essential differences in the type of data and the method used for the analysis compared to our work. Najafabadi et al initially clustered the expression patterns using the "average" across several conditions in expression levels and expression "patterns" to perform the codon usage analysis and tRNA modulation. In our approach, we initially used the codon usage as a mean to identify sets of similar genes and performed the analysis using transcriptome and proteome levels independently for each of the considered conditions.

The initial analysis aimed to identify classes of genes with similar codon usage in their primary sequence using the whole annotated genome. From the SOM analysis we obtained a set of 20 different clusters in which the biggest cluster contained 712 ORFs, and the smallest 190 ORF's. The distribution of the clusters is shown in Figure 3.

**Figure 4:**
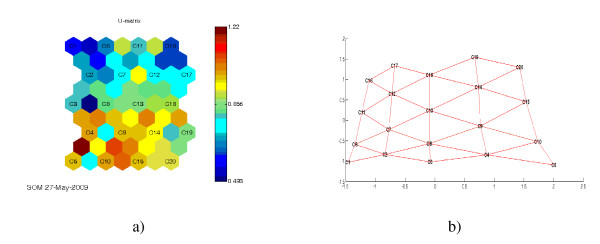
**a) U matrix with the 20 clusters (from C1-C20) and b) PCA-like projection**. SOM clustering was based on the protein amino acid sequences. In the U-Matrix blue color separate neurons that are near to one another, and red to neurons that are distant from one another.

The results of applying SOM can be observed in Figure 4 which contains the unified distance matrix (U-matrix) showing the distances between clusters and also contains the PCA-like projection of the different clusters. Figure 4a) shows the distribution of the clusters and the distances between them. In the PCA-like projection, Figure 4b), it is shown that the separation of the clusters is uniform.

Each of the clusters contains a different number of genes (Figure 3) and to identify the functionality of these genes we applied a hypergeometric distribution test to assess the overrepresentation GO biological process. The BINGO tool [29], a Cytoscape plug in [42], was used to perform the analysis. In total the hypergeometric test reported 596 different GO biological process terms, out of which only 115 were repeatedly observed across the different clusters. The analysis shows enrichment of many terms, and by taking the 5 most significant GO terms (with a p-value < 0.01 and after multiple testing correction, FDR) we observed that there are few overlaps across clusters (see Table 1). The detailed GO analysis is contained in Additional file 3. This observation suggests that the primary structure of proteins can be naturally selected so that the proteins performing similar functions have similar codon frequencies [15,25,43]. The reason for that could be that proteins with similar codon frequencies respond in a similar way to changes in the transcription levels; as it was suggested also in Akashi H. (2003) and Tuller et al. (2007).

**Table 1:** List of GO biological process terms in each cluster after overlap the results from all datasets.

Cluster 1	translation	biosynthetic process	cellular biosynthetic process	cellular protein metabolic process	protein metabolic process
**Cluster 2**	Transport	establishment of localization	localization	transmembrane transport	glutamine family amino acid catabolic process

**Cluster 3**	amine transport	establishment of localization	amino acid transport	transmembrane transport	carboxylic acid transport

**Cluster 4**	GPI anchor biosynthetic process	GPI anchor metabolic process	phosphoinositide biosynthetic process	lipoprotein metabolic process	lipoprotein biosynthetic process

**Cluster 6**	small molecule metabolic process	small molecule biosynthetic process	carboxylic acid metabolic process	oxoacid metabolic process	organic acid metabolic process

**Cluster 7**	small molecule metabolic process	small molecule biosynthetic process	cellular nitrogen compound biosynthetic process	fatty acid catabolic process	organic acid catabolic process

**Cluster 8**	telomere maintenance via recombination				

**Cluster 10**	telomere maintenance via recombination				

**Cluster 11**	small molecule metabolic process	small molecule biosynthetic process	heterocycle metabolic process	cellular nitrogen compound biosynthetic process	cellular ketone metabolic process

**Cluster 12**	endocytosis				

**Cluster 13**	transposition, RNA-mediated	transposition	cellular process	loss of chromatin silencing	cofactor biosynthetic process

**Cluster 14**	transposition, RNA-mediated	transposition	regulation of biological process	regulation of cellular process	protein amino acid phosphorylation

**Cluster 16**	ribosome biogenesis	ribonucleoprotein complex biogenesis	rRNA metabolic process	rRNA processing	ncRNA processing

**Cluster 17**	cellular component biogenesis	nucleic acid metabolic process	macromolecular complex subunit organization	ribonucleoprotein complex biogenesis	RNA metabolic process

**Cluster 18**	nucleic acid metabolic process	cellular response to stress	cellular component organization	nucleobase, nucleoside, nucleotide and nucleic acid metabolic process	response to DNA damage stimulus

**Cluster 19**	cell cycle	cell cycle process	nucleic acid metabolic process	cellular component organization	cell cycle phase

**Cluster 20**	regulation of biological process	biological regulation	M phase	regulation of cellular process	cell cycle phase

*the genes in clusters 5, 9 and 15 were annotated to the GO term "biological process unknown"

Each cluster obtained from the SOM analysis contains genes that show similar codon frequencies. Thus, in order to investigate how much of the variance in the relationship between protein and mRNA fold change is the result of the differences in codon frequency, we estimated the amplification factor *x*_*j *_for each data point according to Eq. 9. The calculations were performed for each of the 6 considered datasets. Table 2 presents the sums of squares of the deviations from the average (Equations 9-13) between and within clusters. It can be seen that for all the datasets, the sum of squares between clusters is higher than the sum of squares within the clusters. For instance, for Usaite.snf1, the fraction of the variability within the clusters is 0.27 and the fraction of variability between the clusters is 0.73. This means that more similar proteins in terms of codon frequency, show similar responses in protein concentration to changes in mRNA, therefore most of the variability in the mRNA-protein relation can be explained by the codon frequency. The rest of the variability is attributed to factors such as protein degradation and seems to be lower compared to the effect of variability in the codon frequency. The F-test shows that except for one out of six datasets, the null hypothesis (e.g. all the clusters have the same average amplification factor) can be safely rejected.

**Table 2:** The variance of the amplification factor in each cluster.

	Usaite.snf1	Usaite.snf4	Usaite.snf1.4	Griffin	Ideker	Washburn
**Within/Total**	0.27	0.09	0.27	0.13	0.39	0.20

**Between/Total**	0.73	0.91	0.73	0.87	0.61	0.80

**F-test (B/W)**	2.70	10.06	2.75	6.63	1.54	4.09

**p-value**	0.001	1E-06	4.5E-5	0.015	0.55	2E-5

Alternatively to this analysis, we used exactly the same procedure but using amino acid content instead of codon frequency. In Additional File 1 the Table 2 presents the values of the variance comparing amino acid content and codon frequency. As it was expected, the same conclusions can be extracted both using codon frequency and amino acid content.

## Conclusions

Experimentally, it has been observed that the correlation between transcriptome and proteome is positive but not high enough to predict protein levels based on their cognate mRNA transcript levels. In this work, by using experimental transcriptome and proteome data together with a statistical analysis, it was shown that most of the variability in the correlation between protein and mRNA concentration can be explained by the differences in codon usage. Thus, genes with similar codon frequencies show similar correlations between mRNA and protein levels. It was also observed that genes involved in the same cellular functions tend to have more similar codon frequencies. A possible explanation for this fact is the evolutionary advantage that would suppose that the concentrations of proteins involved in the same processes respond in similar ways to perturbations in the mRNA levels.

## Authors' contributions

RO and SB developed the method and the mathematical framework. RO performed the data analysis. JN initiated, supervised and coordinated the project. All the authors wrote the manuscript and approved the final version.

## Supplementary Material

Additional file 1 **Description and references for the experimental datasets and comparative table for variances in amino acid content**. Supplementary Table S1. This is the list of the six datasets thet were used in this analysis containing expression values for protein and transcript. These datasets have been published on previous works and are considered as high quality data. Supplementary Table S2. It contains the variance in the amplification factor in clusters built using amino acid content and codon usage respectively. Click here for file

Additional file 2 **Histograms and box plots of the experimental data**. This file contains the histograms and boxplots showing the experimental distributions of the amplification factor, used in the analysis. Click here for file

Additional file 3 **Cluster results and amplification factors data**. This workbook contents the cluster number for each of the ORF annotated for Saccharomyces cerevisiae. The clusters were constructed using the codon sequence content which was normalized suing the total number of codons. Click here for file

## References

[B1] Nielsen J Jewett MC Impact of systems biology on metabolic engineering of Saccharomyces cerevisiae FEMS Yeast Res 2008 8 1 122 131 10.1111/j.1567-1364.2007.00302.x 17727659

[B2] Futcher B Latter GI Monardo P McLaughlin CS Garrels JI A sampling of the yeast proteome Mol Cell Biol 1999 19 11 7357 7368 10523624 10.1128/mcb.19.11.7357 PMC84729

[B3] Gygi SP Rochon Y Franza BR Aebersold R Correlation between protein and mRNA abundance in yeast Mol Cell Biol 1999 19 3 1720 1730 10022859 10.1128/mcb.19.3.1720 PMC83965

[B4] Lu P Vogel C Wang R Yao X Marcotte EM Absolute protein expression profiling estimates the relative contributions of transcriptional and translational regulation Nat Biotechnol 2007 25 1 117 124 10.1038/nbt1270 17187058

[B5] Kudla G Murray AW Tollervey D Plotkin JB Coding-sequence determinants of gene expression in Escherichia coli Science 2009 324 5924 255 258 10.1126/science.1170160 19359587 PMC3902468

[B6] Mehra A Lee KH Hatzimanikatis V Insights into the relation between mRNA and protein expression patterns: I. Theoretical considerations Biotechnol Bioeng 2003 84 7 822 833 10.1002/bit.10860 14708123

[B7] Nie L Wu G Culley DE Scholten JCM Zhang W Integrative Analysis of Transcriptome and Proteomic Data: Challenges, Solutions and Applications Critical Reviews in Biotechnology 2007 27 63 75 10.1080/07388550701334212 17578703

[B8] Mehra A Hatzimanikatis V An algorithmic framework for genome-wide modeling and analysis of translation networks Biophys J 2006 90 4 1136 1146 10.1529/biophysj.105.062521 16299083 PMC1367265

[B9] Zouridis H Hatzimanikatis V A model for protein translation: polysome self-organization leads to maximum protein synthesis rates Biophys J 2007 92 3 717 730 10.1529/biophysj.106.087825 17098800 PMC1779991

[B10] Zouridis H Hatzimanikatis V Effects of codon distributions and tRNA competition on protein translation Biophys J 2008 95 3 1018 1033 10.1529/biophysj.107.126128 18359800 PMC2479585

[B11] Gustafsson C Govindarajan S Minshull J Codon bias and heterologous protein expression Trends Biotechnol 2004 22 7 346 353 10.1016/j.tibtech.2004.04.006 15245907

[B12] Sharp PM Li WH The codon Adaptation Index--a measure of directional synonymous codon usage bias, and its potential applications Nucleic Acids Res 1987 15 3 1281 1295 10.1093/nar/15.3.1281 3547335 PMC340524

[B13] dos Reis M Savva R Wernisch L Solving the riddle of codon usage preferences: a test for translational selection Nucleic Acids Res 2004 32 17 5036 5044 10.1093/nar/gkh834 15448185 PMC521650

[B14] Najafabadi HS Goodarzi H Salavati R Universal function-specificity of codon usage Nucleic Acids Res 2009 37 21 7014 7023 10.1093/nar/gkp792 19773421 PMC2790905

[B15] Tuller T Kupiec M Ruppin E Determinants of protein abundance and translation efficiency in S. cerevisiae PLoS Comput Biol 2007 3 12 e248 10.1371/journal.pcbi.0030248 18159940 PMC2230678

[B16] Tuller T Waldman YY Kupiec M Ruppin E Translation efficiency is determined by both codon bias and folding energy Proc Natl Acad Sci USA 2010 107 8 3645 3650 10.1073/pnas.0909910107 20133581 PMC2840511

[B17] Greenbaum D Colangelo C Williams K Gerstein M Comparing protein abundance and mRNA expression levels on a genomic scale Genome Biol 2003 4 9 117 10.1186/gb-2003-4-9-117 12952525 PMC193646

[B18] Sonenberg N Dever TE Eukaryotic translation initiation factors and regulators Curr Opin Struct Biol 2003 13 1 56 63 10.1016/S0959-440X(03)00009-5 12581660

[B19] Kapp LD Lorsch JR The molecular mechanics of eukaryotic translation Annu Rev Biochem 2004 73 657 704 10.1146/annurev.biochem.73.030403.080419 15189156

[B20] Fluitt A Pienaar E Viljoen H Ribosome kinetics and aa-tRNA competition determine rate and fidelity of peptide synthesis Comput Biol Chem 2007 31 5-6 335 346 10.1016/j.compbiolchem.2007.07.003 17897886 PMC2727733

[B21] Lee SB Bailey JE Analysis of growth rate effects on productivity of recombinant Escherichia coli populations using molecular mechanism models. Reprinted from Biotechnology and Bioengineering, Vol. 26, Issue 1, Pages 66-73 (1984) Biotechnol Bioeng 2000 67 6 805 812 10.1002/(SICI)1097-0290(20000320)67:6<805::AID-BIT16>3.0.CO;2-0 10699859

[B22] McAdams HH Arkin A Simulation of prokaryotic genetic circuits Annu Rev Biophys Biomol Struct 1998 27 199 224 10.1146/annurev.biophys.27.1.199 9646867

[B23] McAdams HH Arkin A Stochastic mechanisms in gene expression Proc Natl Acad Sci USA 1997 94 3 814 819 10.1073/pnas.94.3.814 9023339 PMC19596

[B24] Heyd A Drew DA A mathematical model for elongation of a peptide chain Bull Math Biol 2003 65 6 1095 1109 10.1016/S0092-8240(03)00076-4 14607290

[B25] Lithwick G Margalit H Hierarchy of sequence-dependent features associated with prokaryotic translation Genome Res 2003 13 12 2665 2673 10.1101/gr.1485203 14656971 PMC403808

[B26] Vesanto J Himberg J Alhoniemi E Parhankangas J SOM toolbox 2.0 for Matlab 2005

[B27] Tamayo P Slonim D Mesirov J Zhu Q Kitareewan S Dmitrovsky E Lander ES Golub TR Interpreting patterns of gene expression with self-organizing maps: methods and application to hematopoietic differentiation Proc Natl Acad Sci USA 1999 96 6 2907 2912 10.1073/pnas.96.6.2907 10077610 PMC15868

[B28] Mangiameli P Chen SK West D A comparison of SOM neural network and hierarchical clustering methods European Journal of Operational Research 1996 93 2 402 417 10.1016/0377-2217(96)00038-0

[B29] Maere S Heymans K Kuiper M BiNGO: a Cytoscape plugin to assess overrepresentation of gene ontology categories in biological networks Bioinformatics 2005 21 16 3448 3449 10.1093/bioinformatics/bti551 15972284

[B30] Mei-Ling TL Analysis of Microarray Gene Expression Data 2004 Springer US

[B31] Griffin TJ Gygi SP Ideker T Rist B Eng J Hood L Aebersold R Complementary profiling of gene expression at the transcriptome and proteome levels in Saccharomyces cerevisiae Mol Cell Proteomics 2002 1 4 323 333 10.1074/mcp.M200001-MCP200 12096114

[B32] Ideker T Thorsson V Ranish JA Christmas R Buhler J Eng JK Bumgarner R Goodlett DR Aebersold R Hood L Integrated genomic and proteomic analyses of a systematically perturbed metabolic network Science 2001 292 5518 929 934 10.1126/science.292.5518.929 11340206

[B33] Washburn MP Koller A Oshiro G Ulaszek RR Plouffe D Deciu C Winzeler E Yates JR Protein pathway and complex clustering of correlated mRNA and protein expression analyses in Saccharomyces cerevisiae Proc Natl Acad Sci USA 2003 100 6 3107 3112 10.1073/pnas.0634629100 12626741 PMC152254

[B34] Usaite R Wohlschlegel J Venable JD Park SK Nielsen J Olsson L Yates JR Iii Characterization of global yeast quantitative proteome data generated from the wild-type and glucose repression saccharomyces cerevisiae strains: the comparison of two quantitative methods J Proteome Res 2008 7 1 266 275 10.1021/pr700580m 18173223 PMC2538956

[B35] Usaite R Jewett MC Oliveira AP Yates JR Olsson L Nielsen J Reconstruction of the yeast Snf1 kinase regulatory network reveals its role as a global energy regulator Mol Syst Biol 2009 5 319 10.1038/msb.2009.67 19888214 PMC2795470

[B36] Ghaemmaghami S Huh WK Bower K Howson RW Belle A Dephoure N O'Shea EK Weissman JS Global analysis of protein expression in yeast Nature 2003 425 6959 737 741 10.1038/nature02046 14562106

[B37] Brockmann R Beyer A Heinisch JJ Wilhelm T Posttranscriptional expression regulation: what determines translation rates? PLoS Comput Biol 2007 3 3 e57 10.1371/journal.pcbi.0030057 17381238 PMC1829480

[B38] Nie L Wu G Zhang W Correlation between mRNA and protein abundance in Desulfovibrio vulgaris: a multiple regression to identify sources of variations Biochem Biophys Res Commun 2006 339 2 603 610 10.1016/j.bbrc.2005.11.055 16310166

[B39] Nie L Wu G Zhang W Correlation of mRNA expression and protein abundance affected by multiple sequence features related to translational efficiency in Desulfovibrio vulgaris: a quantitative analysis Genetics 2006 174 4 2229 2243 10.1534/genetics.106.065862 17028312 PMC1698625

[B40] Lithwick G Margalit H Relative predicted protein levels of functionally associated proteins are conserved across organisms Nucleic Acids Res 2005 33 3 1051 1057 10.1093/nar/gki261 15718304 PMC549420

[B41] Welch M Govindarajan S Ness JE Villalobos A Gurney A Minshull J Gustafsson C Design parameters to control synthetic gene expression in Escherichia coli PLoS One 2009 4 9 e7002 10.1371/journal.pone.0007002 19759823 PMC2736378

[B42] Shannon P Markiel A Ozier O Baliga NS Wang JT Ramage D Amin N Schwikowski B Ideker T Cytoscape: a software environment for integrated models of biomolecular interaction networks Genome Res 2003 13 11 2498 2504 10.1101/gr.1239303 14597658 PMC403769

[B43] Akashi H Translational selection and yeast proteome evolution Genetics 2003 164 4 1291 1303 12930740 10.1093/genetics/164.4.1291 PMC1462678

