# Supplementary material for: Codon usage variability determines the correlation between proteome and transcriptome fold changes
Source: BMC Syst Biol. 2011 Feb 25;5:33. doi: 10.1186/1752-0509-5-33 (PMC3058016; doi:10.1186/1752-0509-5-33)
Supplement: Additional file 1 — Description and references for the experimental datasets and comparative table for variances in amino acid content. Supplementary Table S1. This is the list of the six datasets thet were used in this analysis containing expression values for protein and transcript. These datasets have been published on previous works and are considered as high quality data. Supplementary Table S2. It contains the variance in the amplification factor in clusters built using amino acid content and codon usage respectively. [file 1752-0509-5-33-S1.DOC]

**Supplementary Table S1**. Summary of the experimental datasets used in this analysis.

| **Author** | **Comparison** | **Number of genes,**  **Pearson correlation (R^2^) ^a^** | **Aim of study** |
| --- | --- | --- | --- |
| Griffin et al.,  2002 | Glucose/Ethanol  (Griffin^b^) | 239, (-0.112) | Compares the growth using two different carbon sources: glucose and ethanol. |
| Ideker et al.  2001 | wt+ga.l/ wt-gal.  (Ideker) | 288, 0.363 | Transcriptomics and proteomic analysis of the regulatory network in galactose utilization |
| Usaite et al.,  2008  and 2009 | Δsnf1/ wt (Usaite.snf1)  Δsnf4/ wt (Usaite.snf4)  Δsnf1snf4/ wt (Usaite.snf1.4) | 1022, 0.637  980, 0.546  638, 0.569 | Reconstruction of the yeast Snf1 regulatory networks. Deletion of genes *SNF1*,*SNF4* and double deletion *SNF1*-*SNF4*. |
|  |  |  |  |
| Washburn et al.,  2003 | YEPD/^15^N minimal media  (Washburn) | 671, 0.204 | Correlation analysis of mRNA and Protein expression in yeast growing in different media |

^a^ The pearson correlation is calculated transforming the data using log2 and with a significance of p<0.0001

^b^ To identify each dataset trough the text we used an ID composed with the last name of the author. For instance, in case of Griffin et al. datasets it is the composition of the last name.

**Supplementary Table S2.**

The variance of the amplification factor in each cluster using amino acid content.

|  | **Usaite.snf1** | **Usaite.snf4** | **Usaite.snf1.4** | **Griffin** | **Ideker** | **Washburn** |
| --- | --- | --- | --- | --- | --- | --- |
| **within/total (AA)** | 0.28 | 0.11 | 0.21 | 0.43 | 0.52 | 0.20 |
| **between/total (AA)** | 0.72 | 0.89 | 0.79 | 0.57 | 0.48 | 0.80 |
| **within/total (CU)** | 0.27 | 0.09 | 0.27 | 0.13 | 0.39 | 0.20 |
| **between/total(CU)** | 0.73 | 0.91 | 0.73 | 0.87 | 0.61 | 0.80 |

(AA) refers to the amino acid content whereas (CU) referrers to codon usage.
