# Supplementary material for: Codon usage variability determines the correlation between proteome and transcriptome fold changes
Source: BMC Syst Biol. 2011 Feb 25;5:33. doi: 10.1186/1752-0509-5-33 (PMC3058016; doi:10.1186/1752-0509-5-33)
Supplement: Additional file 2 — Histograms and box plots of the experimental data. This file contains the histograms and boxplots showing the experimental distributions of the amplification factor, used in the analysis. [file 1752-0509-5-33-S2.DOC]

Additional Figure 2.A. Histograms of the populations (datasets) showing the normal distribution

Usaite.1 Usaite.4 Usaite.1.4

Griffin Ideker Washburn

Additional Figure 2.A. Box plots of the showing the distribution of the populations (datasets). Outliers where removed

Usaite.1 Usaite.4 Usaite.1.4

Usaite.1 Usaite.4 Usaite.1.4
